# Preliminary design of an innovative, simple, and easy-to-build portable ventilator for COVID-19 patients

**DOI:** 10.1007/s41207-020-00163-1

**Published:** 2020-05-06

**Authors:** Badre El Majid, Aboubakr El Hammoumi, Saad Motahhir, Ambar Lebbadi, Abdelaziz El Ghzizal

**Affiliations:** 1 Independent Scientist, Rabat, Morocco; 2 Innovative Technologies Laboratory, EST, SMBA University, Fez, Morocco; 3 Engineering, Systems and Applications Laboratory, ENSA, SMBA University, Fez, Morocco

**Keywords:** Ventilator, COVID-19, Medical hardware

## Abstract

This technical note describes the preliminary design of a simple, easy-to-use, and easy-to-build ventilator with an unique design that can be used for COVID-19 patients in emergencies and to prevent massive loss of life in resource-poor environments. It can be assembled by a nonexpert as a homemade solution, without the need for specific equipment or technology. The proposed system is novel, inexpensive, has a reduced reliance on external power, and is very easy to maintain.

## Introduction

Respiratory diseases and failures are a major public health issue in both developed and developing countries. This global issue has been greatly accentuated by the COVID-19 pandemic, which has resulted in an urgent need for extra ventilators (Tian et al. 2020). Even developed countries such as Spain, Italy, and the United States are suffering from a shortage of these expensive respiratory devices which also require a relatively long time to manufacture them (Folmer 2020).

There are two types of ventilator devices. One type simply pushes a certain volume of air into the lungs mechanically without accounting for whether the patient wants to draw air into their body or to push air out. Almost all of these devices are based on the use of the conventional bag valve mask (BVM) (Hess and Kacmarek 1996). A BVM is a plastic bag that a clinical care practitioner can deflate manually with their hands, and therefore provides an inexpensive and easy way to force air into the lungs. Indeed, BVMs are applied by first responders to patients who are not breathing, rather than performing mouth-to-mouth resuscitation. All ventilator devices based on a BVM are essentially robotic arms that squeeze the bag again and again at a set frequency. These devices can be manufactured quickly and in large numbers, but since these ventilators are simply pumps that force air into the patient’s lungs, they can only be used for patients under general anesthesia or those who are near death and have nonfunctional lungs. Applying such a device to a conscious patient would lead to a risk of death through barotrauma, which occurs when the human body is exposed to an inappropriate air pressure (Diaz and Heller 2020).

The second type of ventilator is more advanced. These devices are currently being used in resuscitation units to treat COVID-19 (and other) patients, as these ventilators are intelligent enough to be able to discern if the patient wants to draw in air or push it out and then help the patient to achieve the desired action (Medtronic 2020a). A ventilator of this type has many sensors that interact with the human body, and air is supplied deliberately and accurately to the patient based on the sensor data. As an example, the Puritan Bennett 980 mechanical ventilator, which is a top of the line high-performance ventilator, provides advanced synchrony tools to help the clinician to set the ventilator to adapt to each patient’s individual needs and thus provide the appropriate level of support throughout the breath (Medtronic 2020b). However, such devices are very complicated pieces of machinery, making them very expensive and time-consuming to manufacture in large quantities.

Due to the ventilator shortages induced by the COVID-19 epidemic, the world is currently racing to develop a viable ventilation system that is cheap and easy to make. Many enthusiastic engineers who want to help out are volunteering their expertise to develop a low-cost ventilator that could be built by any suitably adapted manufacturing facility (Pearce et al. 2020; Powers and Miller 2020). The vast majority of the solutions that have been proposed utilize a BVM and are based on a research paper from MIT released in 2010 (Al Husseini et al. 2010). An example is the automated manual resuscitator proposed by the MIT E-Vent Project Team (2020). However, some solutions—such as the unique design we propose in the present paper—are completely new ventilator designs. Various new concepts are circulating on the Internet, but many of them are based on a back and forth movement of the motor, which requires a relatively complex control system. In order to maximize the availability of ventilator devices (and minimize the complexity of their production), we believe that it is important to create a design based on unidirectional motion. Therefore, in this technical note, we propose a new, simple, easy-to-build ventilator design.

The remainder of this paper is structured as follows. The design of the proposed novel ventilator and its unprecedented operating principle are presented in the next section. Challenges posed by this new ventilator concept are then discussed. The final section of this technical note summarizes the work reported here.

## Design concept

**Fig. 1:**
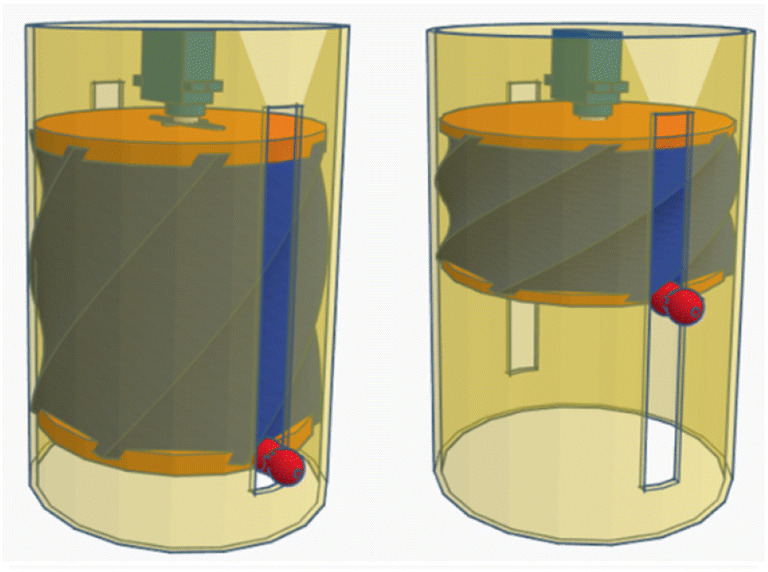
3D view of the assembled ventilator

**Fig. 2:**
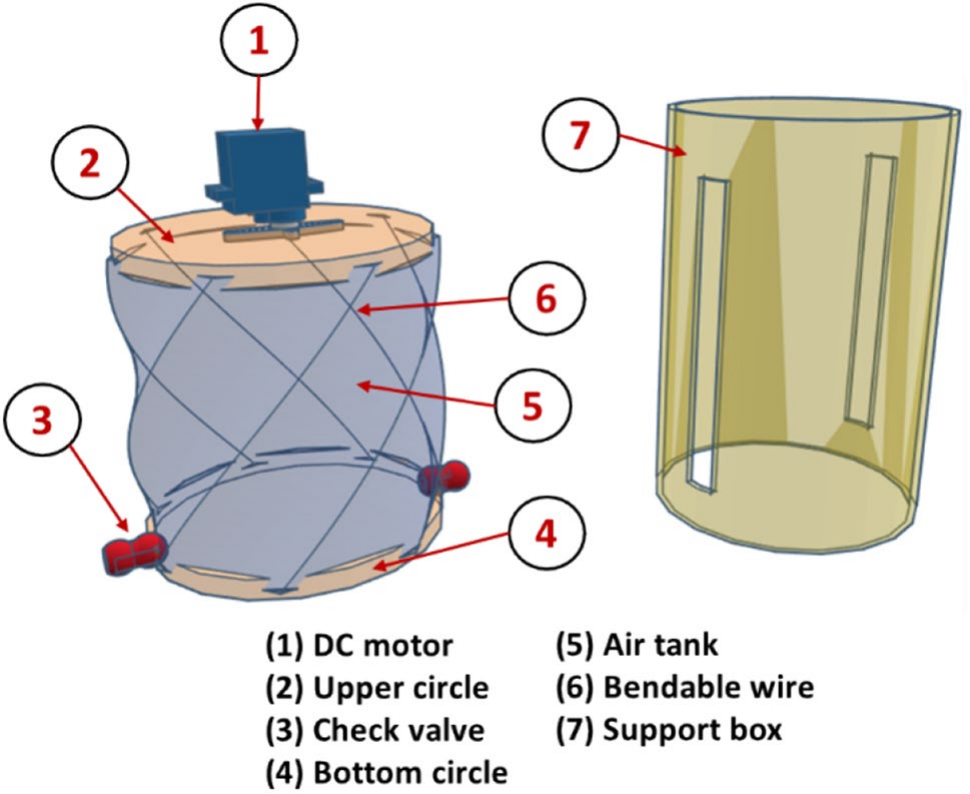
3D view of the contents of the proposed ventilator device

The unique concept of the proposed ventilator is depicted in Figs. 1 and 2. It comprises a plastic air tank, two wooden or plastic circles (fixed and mobile discs), a bendable wire, two check valves, a DC motor, and a support box (guide cylinder).

**Fig. 3:**
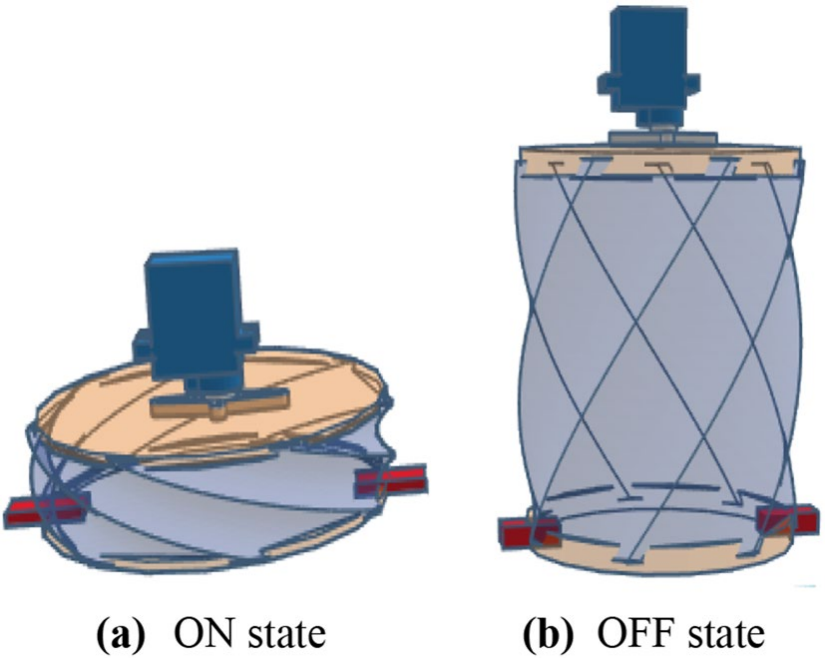
Views of the ventilator in the ON (**a**) and OFF (**b**) states

As shown in Fig. 1, the motor is fixed to the center of the upper circle. In the ON state (Fig. 3a), the motor is activated, causing the upper circle to rotate in one direction. The movement of the motor causes the wire to bend. This pulls the bottom circle upwards, which pressurizes the air inside the tank. This pressurized air is consequently directed into pipes through the check valve. This state corresponds to the inspiration phase.

In the OFF state (Fig. 3b), the motor is released. The bottom circle then moves downwards under the influence of its own weight and the release of the tension in the wire, which is restored to its initial position. Since the pressure in the lungs is higher than the pressure in the air tank, the device will draw air from the patient's lungs. Thus, the OFF state corresponds to the expiration phase. The cycling of ON and OFF states induces respiration. On the other hand, the movement of the bendable wire towards its initial position causes the motor to rotate in the opposite direction, so the motor can be used as a dynamo to generate electricity, thus recharging the battery. This would make our solution less reliant on external power.

Additional components that are not presented in Fig. 2 could also be included to improve our concept. Examples of these components include:

- A temperature sensor (such as the low-cost DS18B20 sensor) on the bottom circle to measure the air temperature
- A heating resistor on the upper circle to control the air temperature
- A pressure sensor (such as the low-cost MPXV5050GP sensor) to measure the pressure in the air tank
- A small rechargeable battery to supply power
- A buzzer to generate audio alarms when, for example, the engine does not work correctly or the battery is low
- Two LEDs (red and green) that indicate the battery status (low or high level of charge)
- A LCD (20 × 4 characters) to display operational data for the system (temperature in the air tank, battery status, etc.)
- A system stop/start button.

All of the components would be controlled by a low-cost embedded board such as an Arduino or ESP32 (a control and data acquisition board).

## Challenges

Based on the promising findings of initial developmental work on the unprecedented ventilator concept presented in this technical note, work is underway to optimize various aspects of the ventilator and to ensure the safety of the patient when the ventilator is operated. More specifically, this work includes:

- Determining the optimal dimensions of the assembly, in particular the diameter of the disc and the height of the air tank according to the maximum air volume required.
- Determining the optimal diameter and material for the bendable wire, as well as the optimal number of wires.
- Choosing an adequate DC motor (torque, speed).
- When torque from the motor is applied to each wire, the distance between the fixed disc and the mobile disc depends on the bending stiffness of the wire. Changing the distance between the discs changes the air volume in the tank. Therefore, it is necessary to perform analytical calculations relating the torque to the wire parameters (e.g., number, diameter, Young modulus, and length).
- The friction between the upper disc and the support must be studied and optimized, as the fit between the upper disc and the support must be tight enough to stop any significant air leakage, but it must not be so tight that it slows down the motor and the ascent of the lower disc during the ON state.
- Adding precise and cost-effective sensors (to measure, e.g., the pressure, volume, and flow) that can provide information employed by the user to control and monitor the tidal volume, inspiratory pressure, bpm, and the inspiratory/expiratory ratio, thus maintaining patient safety and administering air deliberately and accurately.

This novel ventilator system will be developed according to medical standards such as ISO 80601, ISO 5367, and IEC 62304.

## Conclusion

In this technical note, a highly innovative ventilator design is proposed. The concept is still under development. Future iterations will include changes induced by the results of our prototype testing. It will incorporate an adjustable inspiratory/expiratory ratio that will be displayed on a LCD screen. Many cost-effective and precise sensors that interact with the human body and ensure that air is administered deliberately and accurately will also be added. Our goal is to provide a new and inexpensive solution. Finally, we will test the ventilator on various patients' respiratory tracts to compare its performance with conventional ventilators before launching the product onto the market.

## References

[CR1] Al Husseini AM, Lee HJ, Negrete J et al (2010) Design and prototyping of a low-cost portable mechanical ventilator. J Med Devices 4(2):027514 10.1115/1.3442790 PMC7164497 32328214

[CR2] Diaz R, Heller D (2020) Barotrauma and mechanical ventilation. StatPearls, Treasure Island 31424810

[CR10] Folmer K (2020) “We’ll take them all”: demand for ventilators spikes as coronavirus looms [online]. ABC News. https://abcnews.go.com/Health/demand-ventilators-spikes-coronavirus-looms/story?id=69597233. Accessed 10 Apr 2020

[CR4] Hess D, Kacmarek RM (1996) Essentials of mechanical ventilation. McGraw-Hill, New York

[CR5] Medtronic (2020a) Combating COVID-19: Medtronic teams ramp up ventilator manufacturing [online]. https://www.medtronic.com/us-en/about/news/increased-ventilator-manufacture.html. Accessed 9 Apr 2020

[CR8] Medtronic (2020b) Puritan Bennett^TM^ 980 ventilator series [online]. https://www.medtronic.com/covidien/en-us/products/mechanical-ventilation/puritan-bennett-980-ventilator.html. Accessed 9 Apr 2020

[CR3] MIT E-Vent Project Team (2020) Emergency ventilator design [online]. https://e-vent.mit.edu/. Accessed 10 Apr 2020

[CR7] Pearce JM (2020) A review of open source ventilators for COVID-19 and future pandemics [version 1; peer review: 2 approved, 1 approved with reservations]. F1000Research 9:219. 10.12688/f1000research.22942.1 10.12688/f1000research.22942.1 PMC7195895 32411358

[CR6] Powers H, Miller M (2020) Superior Ideas: Innovative Global Solutions Low-Cost Ventilator [online]. https://www.superiorideas.org/projects/igs-low-cost-ventilator. Accessed 10 Apr 2020

[CR9] Tian S, Hu W, Niu L, Liu H, Xu H, Xiao SY (2020) Pulmonary pathology of early-phase 2019 novel coronavirus (COVID-19) pneumonia in two patients with lung cancer. J Thorac Oncol 15:700–704. 10.1016/j.jtho.2020.02.010 10.1016/j.jtho.2020.02.010 PMC7128866 32114094

